# Molecular Epidemiological Investigation of* Porcine kobuvirus* and Its Coinfection Rate with PEDV and SaV in Northwest China

**DOI:** 10.1155/2016/7590569

**Published:** 2016-05-16

**Authors:** Chen Wang, Xi Lan, Bin Yang

**Affiliations:** Lanzhou Veterinary Research Institute, China Academy of Agricultural Sciences, Lanzhou, Gansu 730046, China

## Abstract

*Porcine kobuvirus* (PKV) has circulated throughout China in recent years. Although many studies have detected it throughout the world, its molecular epidemiology has not been characterized in northwest China. To understand its prevalence, 203 fecal samples were collected from different regions of Gansu Province and tested with reverse transcription-polymerase chain reaction. In this study, we tested these samples for PKV, porcine epidemic diarrhea virus (PEDV), and sapovirus and analyzed the amplified 2C gene fragments of PKV. Overall, 126 (62.1%) samples were positive for PKV. Of the 74 piglets samples among the 203 fecal samples, 65 (87.8%) were positive for PKV. PKV infection was often accompanied by PEDV, but the relationship between the two viruses must be confirmed. A phylogenetic analysis indicated that the PKV strains isolated from the same regions clustered on the same branches. This investigation shows that PKV infections are highly prevalent in pigs in northwest China, especially in piglets with symptoms of diarrhea.

## 1. Introduction

A variety of viruses from the large and growing* Picornaviridae* family can infect both humans and animals. Three species of viruses in the genus* Kobuvirus* are now known,* Aichi virus*,* Bovine kobuvirus*, and* Porcine kobuvirus* (PKV) [1]. PKV was discovered in Hungary and China in 2007 [2, 3] and has been detected and isolated in Thailand [4], Japan [5], Korea [6], Brazil, and Netherlands [7].

**Figure 1:**
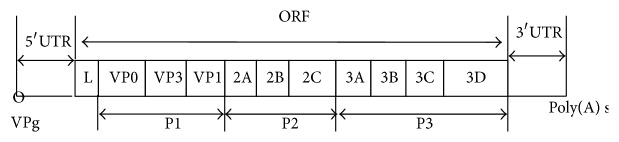
Genomic organization of the kobuviruses.

The genome of this genus consists of a single-stranded positive-sense RNA, ranging from 8.2 to 8.4 kb, including the poly(A) tail [8]. All kobuviruses share essentially the same genomic organization. Nonstructural protein L (leader) is encoded at the N-terminus of the polyprotein and is followed by three structural capsid proteins, VP0, VP3, and VP1 and seven nonstructural proteins, 2A, 2B, 2C, 3A, 3B, 3C, and 3D [9] (Figure 1).

PKV is a virus that may associate with acute viral gastroenteritis in pigs, especially piglets. Its infection rate is relatively high, especially in pigs that are housed. PKV has been reported in many regions of China, including Shanghai City [10], Jiangsu Province [11], Hebei Province [3], Jiangxi Province [12], and Sichuan Province [13]. However, there are no reports of a PKV epidemic in Gansu Province of China to date. In order to better understand the virus prevalence in Gansu Province, PKV and coinfecting pathogens were detected by RT-PCR, and genetic characterization of PKV isolates was performed.

## 2. Materials and Methods

### 2.1. Ethics Statement

The pigs, from which fecal samples were collected, were handled in strict accordance with the Good Animal Practice requirements of Lanzhou Veterinary Research Institute, Chinese Academy of Agricultural Sciences. This study was approved by the Animal Ethics Procedures and Guidelines of the People's Republic of China.

### 2.2. Experimental Protocols

**Figure 2:**
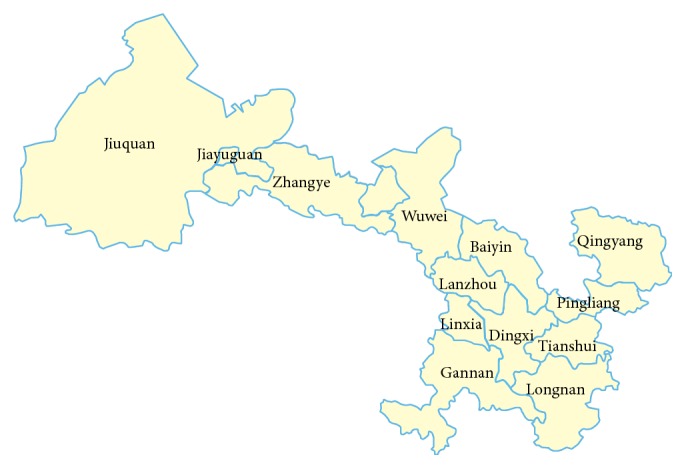
Map of Gansu Province.

A total of 203 porcine fecal samples were collected from the seven main geographic regions of Gansu Province: Lanzhou, Baiyin, Wuwei, Dingxi, Pingliang, Jiayuguan, and Jiuquan (Figure 2). Sampling was representative of the regions of Gansu Province with high numbers of porcine livestock. To determine the groups that are more susceptible to PKV, the 203 fecal samples were divided into two groups: 129 adult pigs and 74 piglets. The piglets are younger than 21 days and the others are older, and samples collected from piglets are all with symptoms of diarrhea; samples collected from adults pigs are not so. Many pathogens cause diarrhea in pigs, including PEDV, RV, TGEV, and SaV, but PEDV, PKV, and SaV are the main etiological factors in pigs detected in Gansu Province. To determine the prevalence of PKV and its relationships with other viruses, we used RT-PCR assays to detect PKV, PEDV, and SaV in all the samples, and constructed a phylogenetic tree using the amplified fragments of the 2C gene of PKV with the maximum-likelihood method.

### 2.3. Primer Design

To detect the presence of PKV, PCR primers were designed based on the sequences of the standard* Kobuvirus* strain S-1-HUN and other strains whose sequences were available from GenBank, using the Primer Premier 6.0 and oligo6 software. The primers target a 544 bp region of the 2C gene. According to a report by Liujian et al. (2013), 3D is the most highly conserved region of the PKV genome, so most detection primers are designed to bind to it. However, we found that the sensitivity and repeatability of the PCR with primers based on this region were not as good as the primer we used based on 544 bp sequence of 2C in our actual assay. The possible reason is that the 2C region is highly conserved among some porcine kobuviruses, as reported previously [14]. For PEDV, purified RNA was subjected to one-step RT-PCR to detect a 765 bp segment of the genome using the P1 primer pair. The S1 primer pair was used to detect SaV, with a 845 bp segment of the genome (Table 1).

### 2.4. RNA Extraction and RT-PCR

Fecal suspensions (20%) were prepared in 0.0067 M phosphate-buffered saline, and centrifuged at 12,000 g for 5 min. The RNA was extracted from 400 *μ*L of the fecal suspensions, using the MiniBEST Universal RNA Extraction Kit (Takara, Dalian, China). All the samples were processed according to the manufacturer's instructions. RT-PCR assays were performed to detect the three viruses with the primers described in Table 1. The one-step RT-PCR was performed with the PrimeScript*™* One-Step RT-PCR Kit ver. 2 from Takara.

### 2.5. Sequence Analysis

We analyzed the amplified 2C gene fragments of PKV and constructed a phylogenetic tree based on it using the maximum-likelihood method in MEGA version 5.0.

### 2.6. Correlation Analysis

In this study, no sample was positive for PKV only, but samples were frequently coinfected with PEDV. We used a correlation analysis to determine the correlation between the incidence of PKV and PEDV with SPSS Statistics 22.0.

## 3. Results

### 3.1. Infection Status of the Examined Pigs

PKV was detected in fecal samples collected from different regions of Gansu Province, China. Of the 203 samples collected, 62.1% were positive for PKV, 68% for PEDV, and 8.9% for sapovirus (SaV). Coinfections of PKV and PEDV were found in 59.6% of the pigs, coinfections of PKV and SaV in 7.4%, coinfections of PEDV and SaV in 8.4%, and multiple infections of PKV, PEDV, and SaV in 7.4% (Table 2). Of the 74 samples collected from piglets, PKV was found in 87.8%, PEDV in 94.6%, SaV in 13.5%, PKV and PEDV in 87.8%, PKV and SaV in 10.8%, PEDV and SaV in 13.5%, and PKV, PEDV, and SaV in 10.8% (Table 3). Of the total fecal samples (*n* = 203), 23.15% were free of all three viruses, and of the 74 piglets samples, 2.7% were free of all three viruses.

**Figure 3:**
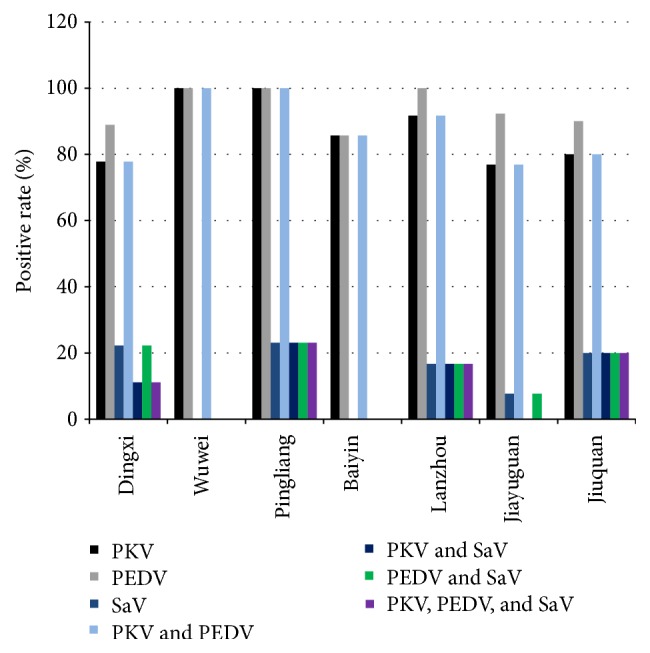
Rates of single infections and coinfections in diarrheal samples from Dingxi, Wuwei, Pingliang, Baiyin, Lanzhou, Jiayuguan, and Jiuquan.

The RT-PCR results indicate that both single-pathogen infections and coinfections with two or three pathogens were more frequent in the piglets samples than in the total samples and that viral prevalence differed in different regions (Figure 3).

### 3.2. Sequencing and Phylogenetic Analysis

To analyze the prevalence of the virus, we used MegAlign to analyze the amplified fragments of the 2C gene of 22 PKV strains (Table 4). The viral samples shared 90.1%–100% nucleic acid sequence similarity. The 15 Gansu isolates shared 90.6%–99.3% nucleotide identity with strain swKoV CH441, which was isolated from northwest China and shared ≥ 90.1% nucleotide sequence identity with strain S-1-HUN. The strains from different regions of Gansu showed sequence variations, suggesting that they have different origins.

**Figure 4:**
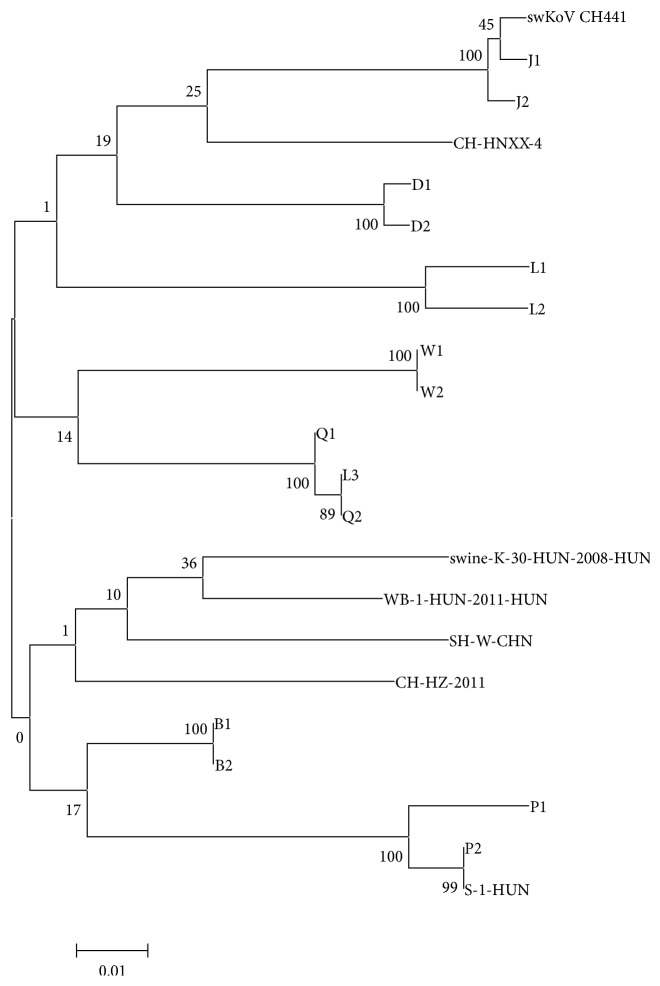
Phylogenetic analysis of the amplified fragments of the 2C gene using the maximum-likelihood method in the MEGA 5.0 software.

On the evolutionary tree, the 15 PKV strains detected in Gansu Province clustered into several groups, and those isolated from the same regions clustered into the same groups (Figure 4). However, those from the Jiuquan samples and sample L3 clustered in a single group, which was an exception to the geographically consistent distributions. Lanzhou is divided into three counties (Yongdeng, Gaolan, and Yuzhong) and five areas (Chengguan, Qilihe, Anning, Xigu, and Honggu). L1 and L2 came from Gaolan, but L3 came from Chengguan. Therefore, the outbreaks of PKV in Jiuquan may be attributable to L3 or vice versa. To avoid the occurrence of a similar situation, we must strictly control the importation and exportation of pigs between regions. The samples from Pingliang and the PKV prototype strain, S-1-HUN, clustered on the same small branch. These results show that the 15 PKV identified in this study may have various genetic relationships with other porcine kobuviruses recorded in GenBank. These findings indicate that PKV displays good host adaption and is endemic to Gansu Province.

### 3.3. Correlation Analysis

A correlation analysis showed that the correlation coefficient between the incidence of PKV and that of PEDV was 0.965. Therefore, PKV may be another pathogen that causes diarrhea in pigs. This conclusion requires further validation.

## 4. Discussion

This study provides insights into the prevalence of PKV infections in Gansu Province. RT-PCR showed that PKV infections were more prevalent in the piglets than in the adult pigs examined, and similar results were observed for PEDV and SaV. And the samples collected from piglets are all with symptoms of diarrhea; samples collected from adults pigs are not so. So kobuvirus is more prevalent in pigs with diarrhea. Besides, PKV infection is often accompanied by PEDV. Since most studies tend to concentrate on a single pathogen, limited data are available on coinfections or mixed infections; the finding that PKV occurs in mixed infections or coinfections with other viruses must be confirmed. Because we also detected PKV in pigs without clinical symptoms, it is possible that this virus is one of the opportunistic pathogens of gastroenteritis in pigs. When pigs are infected with viruses and their immunological resistance is reduced, further infection with PKV will aggravate their condition, hastening death.

In this study, the infection rate of PKV was 62.1% for all the samples with diarrhea collected from Gansu Province, which is higher than the rate of 30.1% in pigs with diarrhea in China [3]. The prevalence of PKV decreased significantly with age, which is consistent with the previous finding that PKV prevalence was highest in pigs <3 weeks old [15].

Kobuviruses have been detected in a variety of animals, including sheep [16], bats [17], dogs [18], goats [19], and cats [20]. However, the full spectrum of kobuvirus hosts must be determined to establish the possibility of cross-species infections among animals and humans and the genomic similarities and homologies between different regions and different hosts.

This study had several weaknesses. The data represent only one specific province of China, the numbers of fecal samples in the different age groups were small, and the sequence used for the evolutionary analysis was short. Therefore, further studies should be conducted to estimate the prevalence of PKV infections in other provinces of China. To avoid the bias caused by the limited numbers of samples or the comparison of sequences of insufficient length, more fecal samples must be collected and their full genomes must be sequenced.

## 5. Conclusion

Piglets, especially those with diarrhea, have a higher prevalence of PKV infection than pigs in all other age groups in Gansu Province. PKV is often present as coinfections or mixed infections with other viruses, including PEDV and SaV. Our findings reveal the high frequency of coinfections or mixed infections of PKV associated with porcine diarrheal diseases and support the need for a national PKV surveillance program in China. What is more, research into the epidemiology of PKV will improve our understanding of this virus, and allow us to control it and ensure the healthy development of animal husbandry in China.

## Figures and Tables

**Table 1:** Sequences of primers.

Primer pair	Sequences of primers (5′ → 3′)	Amplicon length (bp)
K2	K2F: CGTTGGGCTGAGCGTGTA	544
	K2R: AGGGAGCAGAAGAAATGAGGTT	
P1	P1F: AGTCTTACATGCGAATTGACC	765
	P1R: AGCTGACAGAAGCCATAAAGT	
S1	S1F: GCCGTTCACCAGYGTMATAA	845
	S1R: GCCGTTCACCAGYGTMATAA	

**Table 2:** Detection of viruses in the total samples with RT-PCR.

Region	Total samples	Positive samples		
		PKV	PEDV	SaV
Wuwei	39	30	30	3
Lanzhou	25	17	20	2
Dingxi	31	7	8	2
Baiyin	28	20	20	1
Pingliang	32	23	26	7
Jiuquan	22	16	18	2
Jiayuguan	26	13	16	1
Total	203	126	138	18
Positive rate (%)		62.1	68.0	8.9

**Table 3:** Detection of viruses in the piglets samples with RT-PCR.

Region	Total samples	Positive samples		
		PKV	PEDV	SaV
Wuwei	10	10	10	0
Lanzhou	12	11	12	2
Dingxi	9	7	8	2
Baiyin	7	6	6	0
Pingliang	13	13	13	3
Jiayuguan	13	10	12	1
Jiuquan	10	8	9	2
Total	74	65	70	10
Positive rate (%)		87.8	94.6	13.5

**Table 4:** * Kobuvirus* strains analyzed in this study.

Strain	District	Number
W	Wuwei	2
L	Lanzhou	3
D	Dingxi	2
B	Baiyin	2
P	Pingliang	2
J	Jiayuguan	2
Q	Juquan	2
S-1-HUN	Hungary	1
SH-W-CHN	China	1
CH-HZ-2011	China	1
CH-HNXX-4	China	1
swKoV CH441	China	1
WB-1-HUN-2011-HUN	Hungary	1
swine-K-30-HUN-2008-HUN	Hungary	1
