# Corrigendum to “Molecular Epidemiological Investigation of *Porcine kobuvirus* and Its Coinfection Rate with PEDV and SaV in Northwest China”

**DOI:** 10.1155/2017/9794340

**Published:** 2017-08-17

**Authors:** Chen Wang, Xi Lan, Bin Yang

**Affiliations:** State Key Laboratory of Veterinary Etiological Biology, Lanzhou Veterinary Research Institute, Chinese Academy of Agricultural Sciences, Lanzhou, Gansu Province 730046, China

In the article titled “Molecular Epidemiological Investigation of* Porcine kobuvirus* and Its Coinfection Rate with PEDV and SaV in Northwest China” [1] the affiliation of the authors was incomplete. The corrected affiliation is shown above.
